# Angiopoietin-like protein as a novel marker for liver fibrosis in chronic hepatitis B patients with normal to minimally raised ALT

**DOI:** 10.1186/s12879-017-2728-7

**Published:** 2017-09-29

**Authors:** Yongqiong Deng, Hong Zhao, Jiyuan Zhou, Linlin Yan, Guiqiang Wang, China HepB-Related Fibrosis Assessment Research Group

**Affiliations:** 1 Department of Infectious Disease, Center for Liver Disease, Peking University First Hospital, No. 8, Xishiku Street, Xicheng District, Beijing, 100034 China; 2 The Department of dermatology, The Affiliated Hospital of Southwest Medical University, Luzhou, Sichuan China; 3 The Collaborative Innovation Center for Diagnosis and Treatment of Infectious Diseases, Zhejiang University, Zhejiang, Hangzhou China; 4 The coordination innovation centerMinistry of education, Beijing, China

**Keywords:** Angiopoietin-like protein, Hepatitis B, Liver fibrosis

## Abstract

**Background:**

For hepatitis B patients who do not meet the treatment criteria recommended by guidelines, therapy decisions depend on hepatic histology. Angiopoietin-like protein 2 (Angptl2) is a mediator of chronic inflammation that contributes to extracellular matrix remodeling. The aim of this study was to explore the predictive value of Angptl2 as a novel biomarker of liver histology.

**Methods:**

Hepatitis B patients with normal to minimally raised ALT were recruited. Serum Angptl2 concentrations were detected using commercial ELISA kit. The fibrosis score were assessed according to Ishak criteria. Significant fibrosis was defined as ISHAK score ≥ 3.

**Results:**

Of 460 patients, 223 cases served as training cohort and 237 ones as validation cohort. Serum Angptl2 concentration was significantly associated with fibrosis scores in both training and validation group. Angptl2 combined index (ACI) for assessing significant fibrosis was developed from training cohort, based on Angptl2 and conventional variables. ACI showed areas under receiver-operating characteristic curve (AUC) of 0.835 for predicting significant fibrosis, which was superior to APRI (AUC = 0.776, *P* = 0.049), FIB-4 (AUC = 0.750, *P* = 0.010), Hui model (AUC = 0.756, *P* = 0.028), and had a better trend than Forn’s index (AUC = 0.796, *P* = 0.083) in training cohort. Finally, validation cohort revealed its robustness and reliability.

**Conclusion:**

Higher Angptl2 level represents as a potential biomarker independently associated with fibrosis stages. Compared with APRI, Hui model, FIB-4, Forn’s index, ACI did better in diagnosing significant fibrosis in hepatitis B patients.

**Trial registration:**

The complete clinical trials protocol is available by request at clinicaltrials.gov (NCT01962155) and chictr.org (ChiCTR-DDT-13003724).

**Electronic supplementary material:**

The online version of this article (10.1186/s12879-017-2728-7) contains supplementary material, which is available to authorized users.

## Background

Approximately 3.61% of the world’s population (248 million individuals) is chronically infected with hepatitis B virus (HBV), and China, India, and Nigeria are the top three countries with the highest population of HBV surface antigen (HBsAg) positive patients [1]. At least 30% of cirrhosis and 50% of hepatocellular carcinoma (HCC) cases are associated with chronic HBV infection [2]. From an epidemiological survey conducted by the Chinese Center for Disease Control and Prevention in 2006, the weighted prevalence of HBsAg, anti-HBs, and hepatitis B core antigen antibody (anti-HBc) in Chinese individuals aged 1 to 59 years was 7.2, 50.1, and 34.1%, respectively [3]. An accurate initial evaluation of liver damage and the correct treatment decision for this population will not only decrease the future incidence of cirrhosis and HCC but also improve the quality of life and the survival rate. The 2015 APASL guidelines recommend immediately antiviral therapy for HBeAg-positive patients with HBV DNA levels ≥20,000 IU/ml or HBeAg-negative patients with HBV DNA levels ≥2000 IU/ml as well as patients with alanine aminotransferase (ALT) levels greater than 2 times the upper limit of normal (ULN); for patients with ALT <2 × ULN and HBV DNA below thresholds, treatment was suggested if moderate to severe inflammation or significant fibrosis (Ishak fibrosis score ≥ 3) [4]. Although liver biopsy was considered as the reference standard for staging inflammation and fibrosis, it was imperfect due to its invasiveness, sampling bias, inter-observer variations, intra-observer variations, risk of bile leakage, and hemorrhage [5–7]. In the last two decades, a variety of biomarkers and indexes has been developed for non-invasive diagnosis. But few of them were designed for hepatitis B patients with normal to minimally raised aminotransferases, for whom therapy decisions depend on hepatic histology.

Angptl2, a member of the angiopoietin-like protein family, has been recently shown to mediate chronic inflammation and subsequent pathological irreversible tissue remodeling [8]. Angptl2 increases matrix metalloproteinase (MMP) expression and activity through integrin a5ba-mediated activation of P38 mitogen-activated protein kinase (MAPK), thus promoting extracellular matrix (ECM) remodeling [9, 10]. Integrins, which act as functional receptors for Angptl2, are abundantly expressed on macrophages, endothelial cells, and adipocytes [8, 10]. Activated macrophages play a major role in chronic liver disease by synthesizing and releasing a battery of pro-fibrogenic and pro-inflammatory mediators [11]. It reported that angptl2 mRNA was expressed in liver and secreted by hepatocytes [12]. Additionally, higher level of Angptl2 in serum has been found in patients with non-small cell lung cancer, arteriosclerotic occlusion, gastric cancer [13–15], but whether the circulatory concentration of angtpl2 increased in liver disease was unknown. In this study, we hypothesized that Angptl2 participates in hepatic fibrogenesis in chronic liver disease and would be as a potential biomarker for diagnosis of liver fibrosis. The information presented herein might help to improve treatment decision making and avoid liver biopsy in patients with hepatitis.

## Methods

### Patients

Patients aged 18 – 65 years who showed HBsAg positivity for at least 6 months were recruited from 24 centers in Mainland China between October 2013 and May 2016. All patients were treatment naive. Exclusion criteria for this study included concomitant infection with hepatitis C virus (HCV) or human immunodeficiency virus (HIV) and other chronic liver diseases, such as alcoholic liver disease, autoimmune liver disease, heretic liver disease, drug-induced liver disease, and nonalcoholic liver disease. Patients with HCC or decompensate cirrhosis were also excluded. Clinical data, including age, gender, and body mass index (BMI), were recorded at the time of liver biopsy. There were 30 healthy people recruited as control subjects.

### Laboratory tests

Serum hematological and biochemical parameters, such as platelet counts (PLT), ALT, aspartate aminotransferase (AST), albumin, gamma-glutamyltransferase (GGT), prothrombin time (PT), Total bilirubin (TBil) and cholesterol, were routinely obtained within 4 weeks of liver biopsy using standard methodologies in local hospitals. In this study, we are using ALT of 40 IU/ml as the upper limit of the normal [4].

Serum HBsAg levels were quantified using the Roche Elecsys® HBsAg II assay (Roche Diagnostics, Penzberg, Germany), and serum HBV-DNA (dynamic range 2.0 × 10^1−^1.7 × 10^8^ IU/ml) was measured with the COBAS AmpliPrep/COBAS TaqMan method as previously described [16]. The qualitative detection of HBeAg and anti-HBe was also performed using relevant Roche Elecsys® assays according to the manufacturer’s instructions.

The serum concentrations of Collagen IV, laminin (LN), hyaluronic acid, procollagen type III N-terminal peptide (PIIINP) were detected as previously described [17].

Non-invasive indexes for fibrosis, such as APRI, FIB-4, AAR, and Forns’ index, were calculated as follows: APRI = ([AST/ULN]/platelet count[×10^9^/L]) × 100 ; FIB ‐ 4 = (age × AST)/(platelet count)[ × 10^9^/L] × ALT^1/2^) ; Forns^’^ index = 7.811 – 3.131 × LN(Platelet count) + 0.781 × LN(GGT) + 3.467 × LN(Age) − 0.014 × LN(cholesterol) ; Hui model : gx = 1.23 + 0.167 × BMI + 1.191 × (ALP/135) + 0.081 × TBi − 0.139 × Albumin − 0.017 × PLT , Hui model = exp  . (gx)/(1 + exp  . (gx)) [18–21].

### Serum Angptl2 concentration

Serum Angptl2 concentrations were determined using the Human ANGPTL2 Assay kit (Immuno-Biological Laboratories Co., Ltd., Japan) according to the manufacturer’s instructions. To ensure that all samples underwent only one freeze-thaw cycle, the blood samples obtained were used to generate small volume aliquots, which were stored at −80 °C. All Angptl2 tests were performed within 2 weeks. The samples were retested if the coefficient of variation between the duplicate wells was less than 10% or if the R squared value of the standard curve was less than 0.99. In this study, we detected angptl2 concentration in both patients with hepatitis B and 30 healthy people as control.

### Hepatic histological assessment

Ultrasonographic-guided liver biopsies were routinely processed at each institute according to a standardized protocol after receiving the patient’s written informed consent. Specimens were fixed, paraffin-embedded, and stained with hematoxylin-eosin and Masson’s trichrome. A minimum of 2.0 cm of liver tissue with at least 11 portal tracts was required for diagnosis. Pathological interpretations were conducted in the Department of Pathology at the You An Hospital affiliated with Capital Medical University. Each section was blindly and independently assessed by 2 pathologists. When discrepancies occurred, the samples were reviewed by experienced pathologists who were also responsible for reassessment in 10% of randomly selected samples. The strength of concordance was defined by the kappa value. To minimize inter- and intra-observer discrepancies, pre-reading was conducted, and a kappa value ˃0.81 was required. The histological fibrosis stage were assessed according to Ishak criteria [22].

### Statistical analysis

Statistical analyses were performed using SPSS 17.0. Quantitative variables were expressed as the mean ± standard deviation (SD) unless otherwise specified. Categorical variables were compared using Chi-squared tests, and continuous variables were compared using the Kruskal-Wallis and Mann-Whitney U-tests. Spearman’s rank tests were used to study associations between variables and histological scores. Spearman’s correlation coefficient was used as necessary. To determine the independent factors of liver fibrosis, a binary backward stepwise logistic regression analysis was conducted with ISHAK fibrosis stage as the dependent and variables as explanatory variables. Meanwhile the regression coefficient of each independent factors was output at the last step of the binary logistic regression analysis. We calculated a new index gx based on the independent factors and their coefficients, then the model ACI was developed by the formula exp.(gx)/(1 + exp.(gx)). Receiver-operating characteristic (ROC) curves were created for the assessment of variables for staging fibrosis. The performance of variables for predicting the severity of liver damage, expressed as AUC, sensitivity, specificity, positive predictive value (PPV), and negative predictive value (NPV), was calculated. Comparison of ROC curves was performed by MedCalc. All *p* values reported are two sided, and *p* < 0.05 was considered statistically significant.

## Results

### Clinical characteristics of study population

Although there were 685 hepatitis B patients collected, only 460 patients with ALT <2× ULN and qualified biopsy were analyzed in this study. According to the order into the project, 223 cases served as training cohort and 237 ones as validation cohort. Among total patients with normal to minimally raised ALT, one hundred and sixty nine ones showed significant/severe fibrosis (F ≥ 3, 36.8%) (Table 1). In patients with low normal (<0.5 × ULN, *n* = 51), high normal(between 0.5 and 1 × ULN, *n* = 145) and minimally raised ALT (between ULN and 2 × ULN, *n* = 264), Ishak fibrosis score ≥ 3 diagnosed in 13 (25.5%), 55 (37.9%), 100 (38.9%) cases respectively, the data was not shown. Although serum level of AST, Albumin, TBil, PT, Hyaluronic and HBV DNA indicated statistically different between training and validation cohort, fibrosis stages equally distributed in the two sets (*P* = 0.835).

**Table 1:** Baseline Characteristics: Comparison Between the Training and the Validation cohort

Parameter	Training cohort (*n* = 223)	Validation cohort (*n* = 237)	Total (*n* = 460)	*P* value
Age (≥40 year, %)	98 (43.9%)	107 (45.1%)	205 (44.6%)	0.851
Gender (Male, %)	170 (76.2%)	175 (73.8%)	345 (75.0%)	0.591
BMI (≥24 kg/m^2^, %)	82 (36.8%)	84 (35.4%)	166 (36.1%)	0.772
Platelet count (× 10^9^/L)	173.57 ± 56.23	172.42 ± 60.38	172.33 ± 59.08	0.526
ALT (U/L)	44.28 ± 17.13	41.93 ± 17.02	43.07 ± 17.10	0.163
AST (U/L)	35.04 ± 14.58	33.81 ± 16.19	35.50 ± 17.84	0.02
ALP (U/L)	76.06 ± 21.71	78.67 ± 29.75	77.31 ± 26.07	0.736
GGT (U/L)	39.13 ± 43.35	42.76 ± 45.72	41.50 ± 47.42	0.622
Albumin (g/L)	44.45 ± 5.32	44.60 ± 4.98	44.48 ± 5.26	0.039
TBil (μmol/L)	15.65 ± 16.36	19.00 ± 29.98	16.94 ± 22.78	0.037
PT (S)	12.74 ± 1.25	12.36 ± 1.72	12.56 ± 1.49	0.001
HBsAg (log_10_IU/ML)	3.56 ± 0.94	3.57 ± 0.81	3.56 ± 0.88	0.583
Collagen IV (Pg/ML)	936.82 ± 610.24	866.57 ± 472.39	869.97 ± 540.96	0.401
Hyaluronic (μg/L)	124.20 ± 73.76	107.49 ± 68.30	115.26 ± 71.14	0.001
Laminin (μg/L)	94.56 ± 206.58	75.71 ± 145.84	84.24 ± 177.79	0.661
PIIINP (μg/L)	3.59 ± 5.31	3.75 ± 4.86	3.65 ± 5.04	0.442
HBeAg (positive, %)	136 (61.0%)	131 (55.3%)	267 (58.7%)	0.221
HBV DNA (log_10_IU/ML)	6.14 ± 1.93	5.69 ± 2.08	5.89 ± 2.03	0.034
Fibrosis stages (n, %)				0.835
F0	9 (4%)	11 (4.6)	20 (4.3%)	
F1	70 (31.4%)	66 (27.8)	136 (29.6%)	
F2	62 (27.8%)	73 (30.8%)	135 (29.3%)	
F3	40 (17.9%)	45 (19%)	85 (18.5%)	
F4	34 (15.2%)	34 (14.3%)	68 (14.8%)	
F5–6	8 (3.6%)	8 (3.4%)	16 (3.5)	

*BMI* body mass index, *ALT* alanine transaminase, *AST* aspartate transaminase, *ALP* alkaline phosphatase, *GGT* gamma-glutamyltransgerase, *PT* prothrombin time, *HBV*, hepatitis B virus, *HBsAg* HBV surface antigen, *LN* laminin, *PIIINP* Procollagen III N-terminal Peptide

The 30 healthy people aged 18 to 65 years (≥40 year, *n* = 13) were recruited as control subjects, of whom the normal ALT, AST, ALP, GGT, Albumin, TBil, PT and serum HBsAg, HBeAg, HBeAb negativity were required. The HBVDNA level in the control subjects were less than 20 IU/ML. Seventy percent of them were male, and all of them had BMI within 24 kg/m^2^.

### Serum Angptl2 concentration independently associated with significant fibrosis in training group

**Fig. 1:**
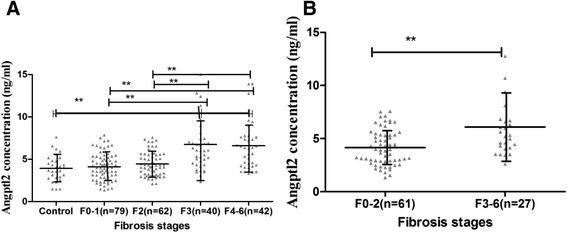
Boxplot of the serum Angptl2 concentrations in relation to fibrosis score in the training cohort all patients (**a**) and patients with normal ALT (**b**) .The above and below lines indicate the SD. The middle line represents the medians. *** *p* < 0.001, **p < 0.01, and *p < 0.05. For all patients in the training cohort, *p* < 0.001. For patients with normal ALT in the training cohort, *p* = 0.003

In 30 healthy people, we detected the Angptl2 concentration of 3.92 ± 1.53 (ng/ml) in serum, which was not different from that in patients with no/moderate fibrosis. However, serum Angptl2 concentration was closely associated with Ishak fibrosis scores for patients in training group, higher the fibrosis stages resulted in higher serum Angptl2 concentrations (*p* < 0.001) (Fig. 1). In patients with normal ALT (*n* = 88), the higher Angptl2 concentration of 6.05 ± 3.94 (ng/ml) was found in 27 (30.7%) patients with significant fibrosis, compared to those with no/moderate fibrosis, who had the Angptl2 level of 4.16 ± 1.66 (ng/ml).

Apart from the higher serum concentrations of Angptl2 in the patients with significant fibrosis, higher levels of Age, AST, ALP, GGT, albumin, PT, hyaluronic, LN, PIIINP, and Collagen IV, as well as lower HBsAg titers and PLT were also found when compared to the other ones with nil/moderate fibrosis. Finally, BMI, age, gender which has been reported to be associated with liver fibrosis and other parameters which showed significantly higher or lower in patients with significant/severe fibrosis (F ≥ 3) were included in multivariate analysis (Table 2). It indicated that serum Angptl2 concentration (*p* = 0.007), PLT (*p* = 0.040), AST (*p* = 0.005), hyaluronic (*p* = 0.002), and LN (p = 0.007) were independently associated with Ishak fibrosis score ≥ 3.

**Table 2:** Variables Association with the Presence of Significant Fibrosis in the Training cohort by Univariate and Multivariate Analyses

Parameters	Univariate analysis			Multivariate analysis	
	F0–2 (*n* = 141)	F ≥ 3 (*n* = 82)	*P*	Exp (B) (95%CI)	*P*
Gender (Male, %)	113 (80.1%)	57 (69.5%)	0.076	1.219 (0.637, 2.332)	0.550
Age (≥35 year, %)	51 (36.2%)	47 (57.3%)	0.03	1.029 (0.988, 1.072)	0.165
BMI (≥24 kg/m^2^, %)	51 (36.2%)	31 (37.8%)	0.886		
Platelet count (× 10^9^/L)	190.68 ± 53.07	142.49 ± 49.17	<0.001	0.990 (0.985, 0.995)	0.001
ALT (IU/L)	42.99 ± 17.28	46.18 ± 16.68	0.159	0.987 (0.969, 1.005)	0.158
AST (IU/L)	31.46 ± 10.24	41.34 ± 18.53	<0.001	1.027 (1.008, 1.047)	0.005
ALP (IU/L)	72.03 ± 19.22	83.55 ± 24.05	0.001	1.006 (0.994, 1.017)	0.339
GGT (IU/L)	27.70 ± 21.92	59.06 ± 61.50	<0.001	1.005 (0.996, 1.013)	0.28
Albumin (g/L)	45.04 ± 4.97	43.27 ± 5.78	0.001	1.027 (0.973, 1.084)	0.334
TBil (μmol/L)	15.41 ± 19.93	16.01 ± 8.11	0.182		
PT (s)	12.54 ± 1.14	13.10 ± 1.38	<0.001	1.067 (0.879, 1.296)	0.509
HBeAg (positive, %)	89 (63.1%)	47 (57.3%)	0.397		
HBV DNA (log_10_IU/ML)	6.43 ± 1.97	5.64 ± 1.77	<0.001	0.782 (0.603, 1.013)	0.063
Collagen IV (Pg/ML)	774.30 ± 302.67	1175.98 ± 887.97	<0.001	1.000 (0.999, 1.002)	0.472
Hyaluronic (μg/L)	97.83 ± 37.96	155.78 ± 86.27	<0.001	1.010 (1.004, 1.016)	0.002
Laminin (μg/L)	32.36 ± 50.72	196.10 ± 312.13	<0.001	1.004 (1.001, 1.006)	0.007
PIIINP (μg/L)	2.97 ± 5.79	4.85 ± 4.98	<0.001	0.968 (0.908, 1.103)	0.312
HBV DNA/HBeAg (n, %)	6.43 ± 1.97	5.64 ± 1.77	<0.001	0.782 (0.603, 1.013)	0.063
Angptl2 (ng/ml)	4.54 ± 1.96	6.07 ± 3.23	0.008	1.152 (1.039, 1.278)	0.007

Based on the variables above, we developed an ACI by binary logistic regression.

$$ \mathrm{Gx}=-1.774+0.027\times \mathrm{AST}-0.01\times \mathrm{PLT}+0.144\times \mathrm{Angptl}2+0.004\times \mathrm{Laminin}+0.009\times \mathrm{Hyaluronic}. $$

$$ \mathrm{ACI}=\exp .\left(\mathrm{gx}\right)/\left(1+\exp .\left(\mathrm{gx}\right)\right). $$

### ACI was compared with APRI, FIB-4, Forn’s index and Hui model for predicting significant fibrosis in training cohort

**Fig. 2:**
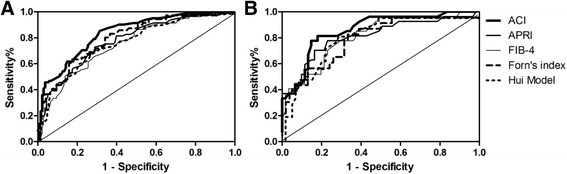
Receiver operating characteristics (ROC) cures of the Angptl2 combined index (ACI), APRI, FIB-4, Forns’ index to distinguish patients with and without significant fibrosis in the Training cohort. **a** Area under the ROC curves (AUC) of above models in the training set. **b** AUC for above models in patients with Normal ALT in the training set

To assess the efficiency of models in predicting significant fibrosis, ROC curves were created (Fig. 2). In the training cohort (223 patients), the AUC of ACI for distinguishing patients who showed significant fibrosis (F ≥ 3) from patients who have no significant fibrosis (F0–2) was 0.835 (95% CI: 0.781, 0.889). Using a cutoff value of ≥0.2, ≥0.3, ≥0.5, patients who need immediate anti-HBV therapy could be correctly identified with a sensitivity of 93.8`, 85.2%, 55.6%, a specificity of 44.0%, 67.2%, 85.8%, a of 50.3%, 61.1%, 70.3%, and a NPV of 92.2%, 88.2%, 76.2% respectively.

Compared to the existing non-invasive assessments, ACI was superior to APRI (AUC = 0.776 (95% CI: 0.713, 0.840)) (*P* = 0.049, Z = 1.962), FIB-4 (AUC = 0.750 (95% CI: 0.684, 0.816)) (*P* = 0.010, Z = 2.571), Hui model (AUC = 0.756 (95% CI: 0.685, 0.827)) (*P* = 0.028, Z = 2.191), and had a better trend than Forn’s index (AUC = 0.796 (95% CI: 0.732, 0.860)) (*P* = 0.083, Z = 1.732) for predicting significant fibrosis (F ≥ 3) in the training group (Fig. 2 A).

Furthermore, predicting value of models for patients with normal ALT were also compared (Fig. 2 B). Similarly, ACI produced AUC of 0.861 (95% CI: 0.778, 0.944) for significant fibrosis, superior to the AUCs of APRI (*P* = 0.063, Z = 1.863), Forn’s index (*P* = 0.023, Z = 2.280), Hui model (*P* = 0.044, Z = 2.016) with 0.811 (95% CI: 0.706, 0.918), 0.792 (95% CI: 0.678, 0.905) and 0.798 (95% CI: 0.685, 0.912), and had a better trend than the AUC of FIB-4 (*P* = 0.089, Z = 1.697) with 0.813 (95% CI: 0.717, 0.909).

### Assessment of noninvasive predictive models in the validation cohort

**Fig. 3:**
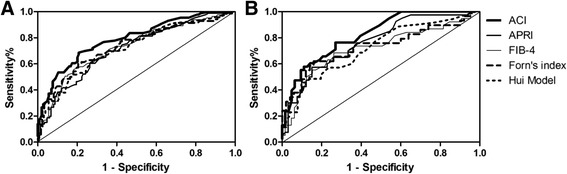
Receiver-operating characteristic curve (ROC) cures of the ACI, APRI, FIB-4, Forns’ index to distinguish patients with and without significant fibrosis in the Validation cohort. (**a**) AUC of above models in the validation set. (**b**) AUC of above models in patients with normal ALT in validation set

Serum Angptl2 concentration showed closely association with Ishak fibrosis scores for patients in training group (Additional file 1: Fig. S1). Diagnostic value of ACI was further assessed together with APRI, FIB-4, Forn’s index, and Hui model (Fig. 3). For predicting significant fibrosis in validation group, the AUROCs were 0.795 (95% CI: 0.730, 0.851) for ACI, 0.715 (95% CI: 0.647, 0.783) for APRI, 0.730 (95% CI: 0.661, 0.799) for FIB-4, 0.735 (95% CI: 0.658, 0.811) for Forn’s index, and 0.719 (95% CI: 0.646, 0.791) for Hui model (Fig. 3A). For patients with normal ALT, the AUC of ACI for predicting significant fibrosis, the indication of urgent anti-HBV therapy was 0.810 (95% CI: 0.727, 0.893), and the AUCs of APRI, FIB-4, Forns’ index and Hui model were 0.766 (95% CI: 0.675, 0.858), 0.717 (95% CI: 0.608, 0.826), 0.732 (95% CI: 0.607, 0.856), 0.721 (95% CI: 0.610, 0.832), respectively (Fig. 3B).

In total 460 patients, predictive accuracy of the models for diagnosing significant fibrosis were calculated, data was shown in Table 3.

**Table 3:** Predictive accuracy of ACI and other index for diagnosing significant fibrosis in total patients

Index	Patients	AUC (95% CI)	Cut-off	Sensiti -vity (%)	Speci-ficit (%)	PPV (%)	NPV (%)	LR
ACI	ALT <2 × ULN (*n* = 460)	0.813 (95% CI: 0.772, 0.854)	≥0.2	88.0	50.9	51.3	90.8	1.6
			≥0.3	76.1	74.4	64.0	84.0	2.97
			≥0.5	50.9	90.0	66.7	74.9	4.80
	Normal ALT (*n* = 196)	0.824 (95% CI: 0.763, 0.884)	≥0.2	81.8	63.7	54.0	87.5	2.25
			≥0.3	65.2	86.3	66.1	84.0	4.75
			≥0.5	40.0	94.4	75.7	75.9	6.98
APRI	ALT <2 × ULN (*n* = 460)	0.742 (95%CI: 0.695, 0.789)	≥0.5	70.0	63.6	53.9	78.8	2.07
			≥1.0	26.4	94.4	73.8	68.9	4.71
	Normal ALT (*n* = 196)	0.780 (95% CI: 0.710, 0.849)	≥0.5	55.2	85.8	58.9	78.3	3.90
			≥1.0	16.4	98.4	84.6	69.1	10.43
FIB-4	ALT <2 ULN (*n* = 460)	0.741 (95% CI: 0.693, 0.788)	≥1.0	89.6	34.2	43.9	66.4	1.42
			≥1.5	17.7	96.4	75.6	67.1	4.97
	Normal ALT (*n* = 196)	0.763 (95% CI: 0.689, 0.830)	≥1.0	90.8	31.8	40.4	86.7	1.33
			≥1.5	21.5	96.8	77.8	70.5	6.78
Forns’ index	ALT <2 × ULN (*n* = 460)	0.766 (95% CI: 0.717, 0.816)	≥6.0	90.1	37.5	44.9	86.8	1.44
			≥8.0	54.6	83.5	64.7	75.8	3.3
	Normal ALT (*n* = 196)	0.762 (95% CI: 0.689, 0.847)	≥6.0	86.54	40.38	41.3	85.1	1.43
			≥8.0	50.0	87.5	67.5	78.4	4.00
Hui Model	ALT <2 × ULN (*n* = 460)	0.736 (95% CI: 0.686, 0.787)	≥0.15	60.1	74.4	58.1	76.5	2.55
			≥0.3	38.5	90.9	71.3	71.7	4.25
	Normal ALT (*n* = 196)	0.745 (95% CI: 0.664, 0.825)	≥0.15	51.8	81.8	58.8	77.4	2.85
			≥0.3	33.9	91.8	66.7	72.7	4.15
			≥0.3	33.9	91.8	66.7	72.7	4.15

## Discussion

The indications for antiviral treatment of hepatitis B were at a long time generally based mainly on the combination of three criteria: serum HBV DNA levels, serum ALT levels and severity of liver disease. According to the treatment criteria of the APASL guideline [4], this 460 patients with ALT <2 × ULN should monitor ALT level every 3 months, biopsy was considered if ALT was persistently elevated, noninvasive tests suggested evidence of significant fibrosis, age > 35 years or family h/o HCC or cirrhosis. In the guideline, although the additional factors were taken into account, the antiviral treatment decision for patients with ALT <2 × ULN still depended on hepatic histology, which is a significantly invasive process.

To date, many publications have demonstrated the growing interest in non-invasive biomarkers and indexes to overcome the limitations of liver biopsy. Individual markers, such as hyaluronate [23], type IV collagen [24], MMPs [25], and CD163 [26, 27], as well as indexes, such as APRI, AAR, FIB-4, Forns’ index and Hui model, have been the most extensively studied. However, the majority of studies investigating non-invasive markers have focused only patients with ALT ≥2 × ULN.

Although ALT levels reflect liver inflammation to some degree, Recent data has increasingly shown that patients with normal or mildly elevated serum ALT levels are not guaranteed to be free from liver damage and liver-related mortality [28]. Twenty eight to thirty seven percent of patients with normal ALT levels were reported to exhibit histologically advanced fibrosis [29–31]. However, few existed serum markers were available that exhibited efficacy for diagnosing significant fibrosis or that aid in treatment decision making in clinical practice, for patients with normal or minimally elevated ALT.In our study, 36.8% of 460 patients who needed biopsies to determine treatment decisions had significant fibrosis. Therefore, non-invasive markers for predicting fibrosis in patients with normal ALT are needed.

This study demonstrated that Angptl2 was significantly associated with fibrosis in patients with normal and minimally elevated ALT. Furthermore, multivariate analysis identified Angptl2 concentration as an independent predictive factor, together with PLT, LN, AST and hyaluronic and developed a model, ACI. To investigate the efficiency of ACI a Angptl2 based model for diagnosing significant fibrosis, we performed further ROC analyses and found it superior to the other 4 existing fibrosis models (APRI, FIB-4, Forns’ index and Hui model). Additionally, validation cohort revealed its robustness and reliability. There was also some problem about standard detection method of angptl2. The circulatory level of Angptl2 in healthy people of different articles changed [13–15], it would be due to measurement by different commercial kit. In this study, the normal level of ANGPTL2 in 30 healthy people was 3.92 ± 1.53 (ng/ml). In the further, the standard kit for testing ANGPTL2 was needed for clinic application.

Normal Angptl2 signaling functions in angiogenesis and tissue repair, whereas excess Angptl2 signaling leads to chronic inflammation and subsequent pathological irreversible tissue remodeling [8]. It was reported that expression of Angptl2 induced by mechanical stress in LF fibroblasts promotes ligamentum flavum (LF) tissue degeneration by activation of TGF-β1/Smad signaling, which resulted in LF hypertrophy in patients with lumbar spinal canal stenosis [32]. Additionally, high levels of Angptl2 protein positively was correlated with histological grade, and liver cirrhosis HCC patients [33]. In this study, Angptl2 exhibited association with liver fibrosis in hepatitis B patients. As discussed above, this may suggests that Angptl2 promote liver fibrogenesis through TGF-β1/Smad signaling, the most potent factor in stimulating collagen gene transcription. The mechanistic studies will be required to clarify the issue.

The current study has limitations that are presented below. First, it is a cross-sectional study. Although a longitudinal study is more powerful in an observational study, most study refer to noninvasive assessment of liver fibrosis are cross-sectional and our multi-center data could be the offset. Second, in this study, normal and minimally elevated ALT is defined within one test and without monitoring it every 3 months. However ALT is just the marker for assessing liver inflammation, but not a marker for diagnosing hepatic fibrosis. Additionally, ALT monitoring always takes time in clinical practice, noninvasive marker superior to ALT to diagnose fibrosis stages could benefit to treatment decision making. The third limitation of our study is clinical research and no mechanism exploration. The basic research of the relationship between Angptl2 and liver fibrosis is currently in progress. Of course, missing imaging examination is also the drawback of this study. The last limitation was that the Angptl2 levels in patients with F3–6 were overlapped with those in patients with F0–2, suggesting the difficulties to set up a cut-off level to estimate the fibrosis stages in chronic hepatitis B patients. So, we developed the ACI model based on Angptl2 together with conventional markers to avoid this problem.

## Conclusion

Although serum Angptl2 concentration needs more extensive validation in the future, the results from this multicenter, prospective study was sufficient to suggest serum Angptl2 concentration as a potentially novel biomarker for predicting the severity of liver injury. In addition, the angptl2 combined index ACI did better in diagnosing significant fibrosis in hepatitis B patients, compared with APRI, Hui model, FIB-4, Forn’s index.

## Additional file

Additional file 1: Fig. S1. Boxplot of the serum Angptl2 concentrations in relation to fibrosis score in Validation cohort (A) and patients with normal ALT (B) .The above and below lines indicate the SD. The middle line represents the medians. *** *p* < 0.001, ** *p* < 0.01, and * *p* < 0.05. For all patients in the validation cohort, *p* < 0.001. For patients with normal ALT in validation cohort, *p* = 0.002. (TIFF 75 kb)

## Abbreviations

AASLD: American Association for the Study of Liver Diseases
ALT: Alanine aminotransferase
Angptl2: Angiopoietin-like protein 2
APASL: the Asian Pacific Association for the Study of Liver
AST: Aspartate aminotransferase
AUC: Area under the curve
BMI: Body mass index
ECM: Extracellular matrix
GGT: Gamma-glutamyltransferase
HAI: Histological activity index
HBsAg: HBV surface antigen
HBV: Hepatitis B virus
HCC: Hepatocellular carcinoma
HCV: Hepatitis C virus
HIV: Human immunodeficiency virus
LN: Laminin
MAPK: Mitogen-activated protein kinase
MMP: Matrix metalloproteinase
NPV: Negative predictive value
PIIINP: Procollagen type III N-terminal peptide
PPV: Positive predictive value
PT: Prothrombin time
ROC: Receiver-operating characteristic curves
SD: Standard deviation
TBil: Total bilirubin
ULN: upper limit of normal
